# Whole brain network effects of subcallosal cingulate deep brain stimulation for treatment-resistant depression

**DOI:** 10.1038/s41380-023-02306-6

**Published:** 2023-11-02

**Authors:** Jungho Cha, Ki Sueng Choi, Justin K. Rajendra, Callie L. McGrath, Patricio Riva-Posse, Paul E. Holtzheimer, Martijn Figee, Brian H. Kopell, Helen S. Mayberg

**Affiliations:** 1 Nash Family Center for Advanced Circuit Therapeutics, Icahn School of Medicine at Mount Sinai, New York, NY USA; 2 Scientific and Statistical Computational Core, National Institute of Mental Health, National Institutes of Health, Bethesda, MD USA; 3 EQRx, Cambridge, MA USA; 4 Department of Psychiatry and Behavioral Sciences, Emory University School of Medicine, Atlanta, GA USA; 5 Department of Psychiatry and Surgery, Geisel School of Medicine at Dartmouth, Lebanon, NH USA

**Keywords:** Depression, Prognostic markers

## Abstract

Ongoing experimental studies of subcallosal cingulate deep brain stimulation (SCC DBS) for treatment-resistant depression (TRD) show a differential timeline of behavioral effects with rapid changes after initial stimulation, and both early and delayed changes over the course of ongoing chronic stimulation. This study examined the longitudinal resting-state regional cerebral blood flow (rCBF) changes in intrinsic connectivity networks (ICNs) with SCC DBS for TRD over 6 months and repeated the same analysis by glucose metabolite changes in a new cohort. A total of twenty-two patients with TRD, 17 [15 O]-water and 5 [18 F]-fluorodeoxyglucose (FDG) positron emission tomography (PET) patients, received SCC DBS and were followed weekly for 7 months. PET scans were collected at 4-time points: baseline, 1-month after surgery, and 1 and 6 months of chronic stimulation. A linear mixed model was conducted to examine the differential trajectory of rCBF changes over time. Post-hoc tests were also examined to assess postoperative, early, and late ICN changes and response-specific effects. SCC DBS had significant time-specific effects in the salience network (SN) and the default mode network (DMN). The rCBF in SN and DMN was decreased after surgery, but responder and non-responders diverged thereafter, with a net increase in DMN activity in responders with chronic stimulation. Additionally, the rCBF in the DMN uniquely correlated with depression severity. The glucose metabolic changes in a second cohort show the same DMN changes. The trajectory of PET changes with SCC DBS is not linear, consistent with the chronology of therapeutic effects. These data provide novel evidence of both an acute reset and ongoing plastic effects in the DMN that may provide future biomarkers to track clinical improvement with ongoing treatment.

## Introduction

Deep brain stimulation (DBS) of the subcallosal cingulate (SCC) region is under active investigation as a potential intervention for treatment-resistant depression (TRD), with repeated past evidence of safety and effectiveness across multiple open-label trials and long-term naturalistic observational studies [1–4]. The therapeutic effects are likely maximized by precisely targeting a network of subcortical and cortical regions connected to or passing through the SCC [5–7]. The critical role of SCC network engagement has been further validated using tissue models combining pre-operative diffusion tractography using diffusion-weighted imaging (DWI) [7, 8] and post-implant stimulation evoked potential mapping using high-density electroencephalogram (EEG) [9].

Clinical reports characterizing the time course of clinical improvement describe rapid and reproducible immediate behavioral effects with initial testing of stimulation in the operating room (OR) [10, 11], and both early and late effects with ongoing chronic stimulation, including a slow progressive improvement in global depression symptoms over weeks to months [4, 10, 12]. In addition, a recent study identified electrophysiological beta power decreases after repeated intraoperative exposure to bilateral therapeutic stimulation that was associated with rapid antidepressant effect that persisted weeks without further stimulation [13]. Neuroimaging studies using positron emission tomography (PET) have previously described widespread changes in limbic and cortical activity measured at 3 and 6 months of ongoing therapeutic stimulation [1, 3]. However, without scans acquired earlier in the course of treatment, there was no opportunity to examine the evolution of these chronic stimulation effects. Chronic treatment with antidepressant medication described differential early and late changes in glucose metabolism at 1 and 6 weeks [14]. Therefore, the brain-wide network change patterns may coincide with the differential early and late effects seen clinically.

In parallel, neuroimaging studies more broadly have discovered that the human brain can be organized into topographically constrained, large-scale intrinsic connectivity networks (ICNs) [15, 16]. Using these well-validated models, resting-state functional magnetic resonance imaging (rs-fMRI) studies of depressed patients have reported abnormalities in several of these ICNs, notably the default mode network (DMN), executive control network (ECN), and salience network (SN), overlapping the location of regional changes identified in the previous DBS PET studies [1, 3, 17–19].

The present study examined the longitudinal brain changes in network regions-of-interest (ROI) defined using these ICNs with SCC DBS, informed by the observed response timeline in recently published reports [4, 7, 8] from two independent cohorts. We assessed changes in regional cerebral blood flow (rCBF) measured using [15 O]-water PET at four-time points: at preoperative baseline, 1-month after surgery, and 1-month and 6-months after chronic therapeutic stimulation. We then examined whether the results would replicate in a new [18 F]-fluorodeoxyglucose (FDG) PET cohort. By combining large-scale ICN ROIs and longitudinal resting-state PET, we evaluated differential changes in the brain’s functional architecture over time and the relationship between specific ICN changes and clinical improvement with ongoing DBS therapy.

## Materials and methods

The two independent DBS trials were performed under physician sponsored Investigational Device Exemptions (FDA IDE G060028 or G130107), (sponsor HSM), and registered in ClinicalTrials.gov (NCT00367003 or NCT01984710). All patients provided written informed consent to participate in the studies. Both protocols were in accordance with the Declaration of Helsinki. The PET studies were approved by the Emory University Institutional Review Board for human research and by the Icahn School of Medicine at Mount Sinai (ISMMS) Institutional Review Board, respectively.

### Participants and lead implantation

Twenty-two patients with TRD receiving bilateral SCC DBS were enrolled from two independent cohorts. The first cohort (cohort 1) consisted of 17 patients (7 men and 10 women) studied at Emory University School of Medicine (Libra DBS device, St. Jude Medical Neuromodulation, Plano Texas), and the second cohort (cohort 2) of 5 patients (2 men and 3 women) studied at ISMMS (Summit RC + S device, Medtronic, Minneapolis MN). Participants from cohort 1 included patient with both unipolar (*n* = 10) and bipolar (n = 7) depression patients; cohort 2 enrolled solely unipolar subjects (*n* = 5). Both unipolar and bipolar depression were combined as done in the previously published clinical report [2]. The surgical target and recruitment criteria were comparable for the two studies and have been previously described [2, 8]. All patients had current major depressive episodes of at least 12 months’ duration and not responding to at least four adequate antidepressant treatments (scoring 3 or higher on the Antidepressant Treatment History Form [20] and verified through medical records) and lifetime failure or intolerance of electroconvulsive therapy (ECT; one patient did not receive ECT), or inability to receive ECT [21]. Mean 17-item Hamilton Depression Rating Scale (HDRS-17) [22] was 23.9 (cohort 1) and 25.25 (cohort 2) averaged over the 4 weeks preceding the implantation surgery. Response was defined as a reduction of 50% in the HDRS-17 at 6 months compared to baseline [23].

For cohort 1, 13 of the 17 patients were implanted awake using anatomical MRI targeting method and tested in the OR with short exposures to 6 mA (starting therapeutic doses) of unilateral DBS delivered to all 8 contacts [2]. Open-label chronic stimulation was initiated after the second PET scan (1-month after surgery). Starting DBS parameters were comparable for all patients (monopolar stimulation, one contact per hemisphere, Frequency=130 Hz, Pulse Width=91 μs, Current=6 mA) with parameters maintained constant for the 1st month. During the 6-month active stimulation phase, 12 of the 17 patients received adjustments, based on the HDRS-17, with increases in current made monthly if there was no clinical improvement. After 6-months of chronic stimulation, 6 patients were receiving 6 mA and 11 patients were receiving 8 mA [2].

For cohort 2, all 5 patients were implanted awake using diffusion tractography targeting method and tested in the OR with short exposures to 6 mA of both unilateral DBS delivered to all 8 contacts as well as bilateral stimulation to the predefined optimal contact. As for cohort 2, open label stimulation was initiated after the second PET scan (2 weeks after surgery) using bilateral monopolar stimulation to a single contact per hemisphere (130 Hz, 90 μs, 4.5 mA). Increases in current could be also made if needed. After 6-months of chronic stimulation, 4 patients were receiving 4.5 mA and 1 patient was receiving 6 mA. No patients had contact changes during the 6 months.

### Image acquisition

The resting-state PET scans were acquired 1 week prior to surgery (preoperative baseline), 1-month (cohort 1) or 2 weeks (cohort 2) after DBS implantation (single-blind placebo OFF period), and after 1 and 6-months of open-label chronic stimulation (stimulation ON).

#### Emory University

Four resting-state [15 O]-water PET scans were acquired at each of four-time points in each of the 17 patients over the course of the DBS experimental trial. In other words, a total of 16 [15 O]-water PET scans (4 scans × 4 timepoints) were acquired for each patient. Scans were collected on a Siemens High-Resolution Research Tomograph (HRRT) scanner (Siemens Medical Solution) using standard methods (matrix size=172 × 172, and number of slices=137) and measured attenuation (low-dose x-ray CT) without arterial blood sampling. The rCBF was measured using the bolus [15 O]-water technique (20 mCi dose/scan; scan duration=60 s) [1]. Scans have spaced a minimum of 11 minutes apart to accommodate radioactive decay to background levels. The second scan of two patients (one responder and one non-responder) was excluded from analysis due to poor image quality.

A high-resolution T1-weighted structural image was collected prior to surgery using a 3 T Siemens Tim Trio scanner (Siemens Medical Solution): magnetization-prepared rapid gradient echo (MPRAGE) sequence, sagittal slice orientation, slice thickness=1 mm, in-plane Resolution=1 mm ×1 mm, matrix=240 × 240, repetition time (TR) = 2600 ms, inversion time (TI) = 900 ms, echo time (TE) = 3.02 ms, and flip angle=8°. Postsurgical high-resolution computed tomography (CT) scan was also acquired on a LightSpeed16 (GE Healthcare) with a resolution of 0.46 × 0.46 × 0.65 mm^3^ to define the location of the DBS leads [7].

#### ISMMS

Glucose metabolism was measured using [18 F]-fluorodeoxyglucose, collected on a Siemens Biograph Vision 600 PET/CT scanner (Siemens Medical Solution). Resting state scans were acquired at the same 4 time points using a standardized eyes closed protocol (10 mCi dose/scan, 30 minutes uptake period without arterial blood sampling, matrix size=440 × 440, and numbers of slice=88). A low-dose CT scan was performed for attenuation correction.

A high-resolution T1-weighted structural image was acquired on a 3 T GE SIGNA Architect (GE Healthcare) using the following sequence: axial slice orientation, slice thickness=1.2 mm, in-plane Resolution = 0.6 mm × 0.6 mm, matrix=256 × 256, TR = 8.432 ms, inversion time (TI) = 1100 ms, echo time (TE) = 3.188 ms, and flip angle=8°. Postsurgical high-resolution CT scan was also acquired on a GE Revolution EVO (GE Healthcare) with a resolution of 0.625 × 0.625 × 0.625 mm^3^ to localize the DBS leads.

### PET image processing

Image processing was performed using Statistical Parametric Mapping 12 (SPM12; http://www.fil.ion.ucl.ac.uk/spm/) and Analysis of Functional NeuroImages (AFNI; http://afni.nimh.nih.gov/afni) software [24] for both datasets, independently. For each CBF session, the four serial 2-minute PET images were first realigned and rigidly co-registered onto the corresponding skull-stripped post-op anatomical CT and pre-op MRI scans. Due to readily visible artifacts near the electrodes, a reversed electrode mask was next created to restrict subsequent analyses to only those brain areas without obvious or potential PET signal corruption. The individual CT image was co-registered to the corresponding structural MR image, to delineate the exact electrode location and define an electrode mask. The electrode mask was then smoothed using a Gaussian kernel with a full-width-half-maximum (FWHM) of 6 mm, binarized, and reversed. In this reversed electrode mask, all voxels immediately adjacent to the electrode along its entire trajectory were assigned a value of 0 and were extracted, with the remaining voxels (value = 1) defining those regions brain-wide without artifact. rCBF counts were lastly scaled proportionally to the total brain radioactivity within the mask. The four normalized rCBF images at each time-point were then averaged. The averaged rCBF maps were warped to the Montreal Neurological Institute (MNI) standard space using the deformation parameters that were previously estimated on T1-weighted image normalization. The normalized rCBF maps were finally smoothed within each reversed electrode mask using AFNI 3dBlurInMask with a Gaussian kernel with a FWHM of 6 mm. The overall data preprocessing and the reversed electrode mask are shown in Supplementary Fig. 1. The same processing was performed on FDG PET images from cohort 2, although there was only one image per time point. Standardized uptake value ratio (SUVR) image was created using the mean global uptake in the reversed electrode mask to estimate the relative glucose metabolism.

### Parcellation of PET data into intrinsic connectivity networks

The PET datasets were anatomically parcellated into 17 standard ICN regions-of-interest (ROI) as defined by a series of published resting-state functional connectivity studies in healthy adults [15]. The location of the 17 ICNs was illustrated onto the cortical surface in Supplementary Fig. 2A, and the details of the parcellation method and naming of the ICNs were described in the previous study [15]. The rCBF for each ICN was calculated by averaging the rCBF values for all voxels within each ICN ROI, excluding those voxels excluded by the previous electrode masking procedure. An 18th ROI was generated to define brain regions with structural connections to the SCC DBS target region. This SCC-DBS depression network ROI (Supplementary Fig. 2B) was defined by a white matter activation pathway template built from these 17 patients in the previous study [7]. The morphologic dilation operator across the 18 voxel neighbors were performed on a template three times and the final ROI was then restricted to the gray matter. For subcortical regions, 16 subcortical ROIs were defined using a previously published resting-state functional connectivity template [25]. Finally, for each patient, the 34 mean values reflected the CBF levels in the regions were obtained. Identical procedures were performed on the FDG PET dataset from cohort 2 for the replication analyses.

### Statistical analyses

The primary analyses focused on the primary CBF dataset. All statistical analyses were conducted using AFNI, MATLAB 2019a (MathWorks Inc., https://www.mathworks.com), and Jamovi (https://www.jamovi.org). The ICN-based rCBF analyses were used rather than voxel-wise analyses to reduce Type I error given the sample size. A linear mixed model approach for the repeated measures (scans at 4 time points) with random intercepts for patient factors was first used to examine the differential trajectory of rCBF changes in the 18 networks (17 standards ICNs + 1 tractography-derived SCC-DBS depression network) over time. The threshold of *q* < 0.05 was set at False Discovery Rate (FDR) to correct for multiple comparisons. Post-hoc contrast tests between all pairs of time points were also performed to characterize differences in changes following initial testing in the OR (Scan2 vs. Scan1), and early (Scan3 vs. Scan2, or Scan1) and late (Scan4 vs. Scan3, or Scan2, or Scan1) changes with ongoing chronic stimulation. Additional, among those ICNs that showed a significant time effect, the relationship of rCBF change and HDRS-17 change relative to the pre-surgical baseline was assessed using a separate linear mixed model. Subsequently, two post-hoc analyses including the changes following initial testing in the OR (Scan2-Scan1) using linear regression, or the changes with ongoing chronic stimulation (Scan3-Scan1 and Scan4-Scan1) using linear mixed model were performed based on the previous clinical report [12].

## Results

**Fig. 1:**
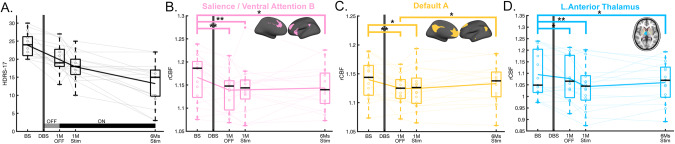
Longitudinal changes of Hamilton Depression Rating Scale (HDRS-17) and regional cerebral blood flow (rCBF) in intrinsic connectivity networks (ICNs). **A** HDRS-17 over time. The gray line in the bottom indicates the off-stimulation phase for 1-month, while the black line in the bottom indicates the chronic stimulation on. **B–D** Time effect of rCBF in ICNs using linear mixed model for repeated measure (FDR q < 0.05). There were time effects in (**B**) salience/ventral attention B (F = 4.62, q = 0.042), (**C**) default A (F = 4.94, q = 0.045), (**D**) left anterior thalamus (F = 5.88, q = 0.032). Asterisk represents statistically significant changes (*: *p* < 0.05, **: *p* < 0.005). Transparent lines in the boxes represent individual longitudinal trajectories, and the solid line is the mean of longitudinal trajectory.

Demographic characteristics and comparisons between groups in the two independent cohorts are available in Table 1. After 6 months of stimulation, 7 patients for cohort 1 and 4 for cohort 2 met antidepressant response criteria. Figure 1A shows the HDRS-17 of each scan for cohort 1 and the percent changes of HDRS-17 from baseline for responders and non-responders was presented in Fig. 4A. Both responders and non-responders showed a common response trajectory for the first month of active stimulation but diverged afterwards. Neither group showed a significant clinical effect in the single-blind placebo month after surgery. Regarding cohort 1, although intraoperative behavioral effects were observed in several patients, these effects exhibited minimal carryover post-operation, and no significant clinical response was evident during the subsequent 1 month of single-blind sham stimulation treatment. On the other hand, in cohort 2, all patients exhibited intraoperative antidepressant effects to bilateral stimulation that decayed over the subsequent month without ongoing stimulation, as reported in a previous study that used the same targeting method (45.6% total decline) [13]. However, it is not possible to disambiguate activity changes due to persistent antidepressant effects from nonspecific post-operative changes that are likely both present 1 week after surgery. The baseline PET scans did not show any significant differences between the unipolar and bipolar depression across all ICNs. In addition, there was a similar trajectory between unipolar and bipolar depression group in responders or non-responders (Supplementary Fig. 3). All patients in cohort 2 show a more considerable reduction of HDRS-17 after 1-month of stimulation than in cohort 1. However, the same diverging pattern after 1 month of active stimulation was observed with responders and non-responders (Fig. 5A).

**Table 1:** Demographic and clinical characteristics of deep brain stimulation patients.

	Emory				ISMMS		
Characteristics	All patients (*n* = 17)	Responder (*n* = 7)	Non-responder (*n* = 10)	Test between responder and non-responder (*p* value)	All patients (*n* = 5)	Responder (*n* = 4)	Non-responder (*n* = 1)
Sex: n_female_ / n_male_	10/7	4/3	6/4	0.906	3/2	2/2	1/0
Age at baseline	42.0 ± 8.92 [27, 58]	43.3 ± 11.6 [27, 58]	41.0 ± 7.02 [33, 55]	0.635	41.0 ± 14.4 [26 60]	40.3 ± 17.6 [26 60]	32
HDRS-17							
Baseline	23.9 ± 3.28 [20, 30]	23.4 ± 3.86 [20, 29]	24.2 ± 2.97 [20, 30]	0.648	25.3 ± 2.6 [22.75 28]	24.6 ± 2.4 [22.75 28]	28
After 1-month of DBS implantation	20.3 ± 3.94 [13, 27]	20.0 ± 4.34 [13, 26]	20.4 ± 3.91 [16, 27]	0.839	18.4 ± 5.6 [12 26]	17.8 ± 6.2 [12 26]	21
After 1-month of chronic stimulation	17.9 ± 3.64 [10, 25]	16.0 ± 3.16 [10, 19]	19.2 ± 3.49 [14, 25]	0.073	10.4 ± 3.3 [7 14]	9.50 ± 3.0 [7 13]	14
After 6-months of chronic stimulation	13.2 ± 5.84 [3, 22]	7.29 ± 3.25 [3, 10]	17.4 ± 2.59 [15, 22]	<0.001	10.2 ± 4.8 [5 18]	8.25 ± 2.22 [5 10]	18

For continuous variables, mean ± standard deviation (SD) [minimum, maximum] is indicated. For comparisons between responder and non-responder, χ2 tests of association were used for discrete variables and *t* tests were used for continuous variables. HDRS-17: 17-items of Hamilton Depression Rating Scale, DBS: deep brain stimulation. Response was defined as a reduction of 50% of the HDRS-17 after 6-months of chronic stimulation relative to the preoperative baseline.

### Longitudinal changes of rCBF in ICNs

A significant time effect was seen for 2 of the 17 ICNs (Fig. 1B, C): salience/ventral attention B (SN: F = 4.62, q = 0.042, Fig. 1B), and default A (DMN: F = 4.94, q = 0.045, Fig. 1C). The SCC-DBS depression network also showed similar decreases to the ICNs, although the changes were not significant after FDR correction (F = 4.79, uncorrected *p* = 0.005) (Supplementary Fig. 2B). For subcortical ROIs, the left anterior thalamus showed a significant time effect (F = 5.88, q = 0.032, Fig. 1D).

Post-hoc analyses revealed a differential trajectory of changes across the ICNs. The ICNs decreases were maximal at 1-month post-surgery relative to baseline (change at 1-month post-surgery: Scan2 vs. Scan1, SN and DMN: *p* = 0.001, Fig. 1B, C). SN decreases were maintained over time with ongoing active stimulation (Fig. 1B), whereas DMN shows a decrease at 1-month post-surgery, but then moderate increase in early (Scan 3 vs. Scan 2) and late (Scan 4 vs. Scan 3) ongoing active stimulation and significant early and late net increase with chronic stimulation (early and late changes: Scan 4 vs. Scan 2, *p* = 0.03, Fig. 1C). For subcortical ROIs, the left anterior thalamus showed significant decreases at 1-month post-surgery (*p* = 0.044), and a maximal decrease with 1-month of active stimulation (Scan3 vs. Scan1, left: p < 0.001) that was sustained over time (Fig. 1D).

### Main effect of clinical improvement

**Fig. 2:**
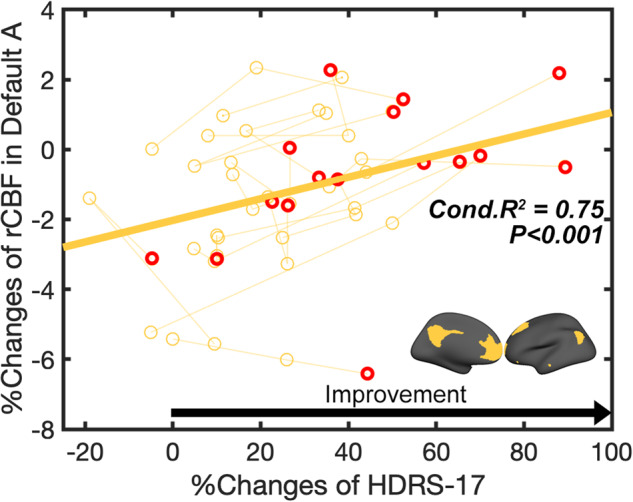
Main effect of clinical improvement. Relationship between percent changes of rCBF and percent changes of HDRS-17 from baseline in default A networks (Conditional R^2^ = 0.75, *p* < 0.001). Red circle indicates the last time point of each patient (after 6-months of chronic stimulation).

There was a significant positive relationship between percent change in the HDRS-17 improvement and percent change in DMN CBF (Fig. 2, Conditional R^2^ = 0.75, p < 0.001). Changes in no other ICN showed this relationship to clinical improvement.

**Fig. 3:**
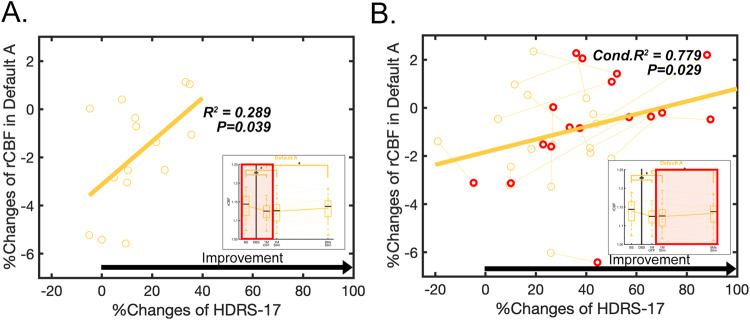
Main effect of clinical improvement after surgery or ongoing chronic stimulation from baseline. **A** Relationship between percent changes of rCBF and percent changes of HDRS-17 after surgery in default A network (R^2^ = 0.289, *p* = 0.039). **B** Relationship between percent changes of rCBF and percent changes of HDRS-17 with ongoing stimulation in default A network (Conditional R^2^ = 0.779, *p* = 0.029). Red circle indicates the last time point of each patient (after 6-months of chronic stimulation).

Post-hoc analyses revealed two significant positive relationships between the percent change in the HDRS-17 and the percent change in DMN CBF: (1) baseline vs. 1-month post-surgery (Fig. 3A, R^2^ = 0.289, *p* = 0.039), even though the DMN decreases were maximal at 1-month post-surgery relative to baseline, (2) ongoing chronic stimulation (Fig. 3B, Conditional R^2^ = 0.779, *p* = 0.029).

### Main effect of time in responders versus non-responders

**Fig. 4:**
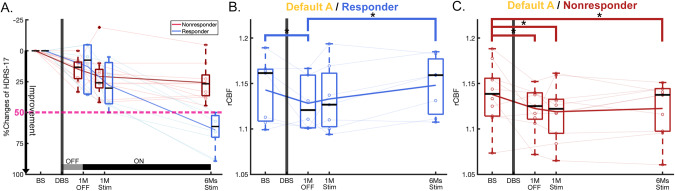
Changes of HDRS-17 and rCBF of default A in responders and non-responders. **A** HDRS-17 over time for responders and non-responders with DBS. **B** Significant rCBF changes over time in default A in responders (F = 3.96, *p* = 0.026). For post-hoc analysis, there were not only early decreased changes of rCBF after surgery (*p* = 0.023), but also late increased rCBF changes between after surgery and 6-months stimulation (*p* = 0.006). **C** Significant rCBF changes over time in default A in non-responders (F = 3.13, *p* = 0.043). For post-hoc analysis, there were early decreased changes of rCBF after surgery (*p* = 0.015) and maintained over time. Asterisk represents statistically significant changes (*: *p* < 0.05).

Both responders and non-responders showed DMN decreases at 1-month post-surgery (Scan2 vs. Scan1, responders: *p* = 0.023, and non-responders: *p* = 0.015) (Fig. 4B, C). The brain effects were not accompanied by significant clinical effects at this time point in either group (1-month post-surgery, without active stimulation; Fig. 4A). Responders, however, showed DMN increases with ongoing chronic stimulation (early and late changes: Scan2 vs. Scan4, *p* = 0.006, Fig. 4B). In contrast, non-responders did not show the changes with chronic stimulation (Fig. 4C).

### Replication using [18 F]-FDG PET

**Fig. 5:**
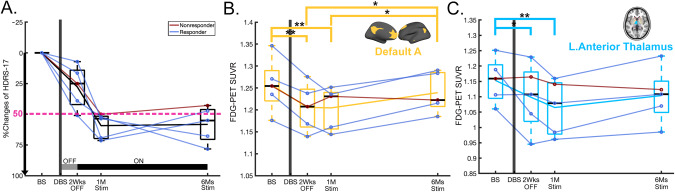
Summary of replicated results in cohort 2 using [F18]-FDG PET. Red line represents the responders (*n* = 4), and blue line represents the non-responders (*n* = 1). **A** Percent changes of HDRS-17 over time from baseline. Longitudinal patterns of standardized uptake value ratio (SUVR) in ICNs which were significant in the cohort 1; (**B**) default A, and (**C**) left anterior thalamus. Asterisk represents statistically significant changes (*: *p* < 0.05, **: *p* < 0.005).

Replicating the CBF findings from cohort 1, a significant time effect was seen in cohort 2 using repeated measures of glucose metabolism using FDG PET scans over 6 months (Fig. 5). Cohort 2 showed similar clinical changes with chronic stimulation to cohort 1, although the magnitude of the changes in HDRS-17 after 1-month of chronic stimulation was greater in cohort 2 (Fig. 5A), consistent with the optimized white matter tractography guided surgical targeting and bilateral stimulation performed in the OR. On the other hand, cohort 1 was anatomically targeted and had only unilateral stimulation in the OR.

Qualitatively, changes in SUVR followed a similar longitudinal pattern as cohort 1 with the involvement of DMN (q = 0.048, Fig. 5B) and left anterior thalamus (q = 0.03, Fig. 5C) after FDR correction, but SN was not significant (q = 0.103). In DMN, all patients showed a significant decrease after surgery (Fig. 5B). However, responders showed an increased SUVR with chronic stimulation (Blue, Fig. 5B), whereas a non-responder showed a decrease in SUVR (Red, Fig. 5B). Regarding the main effect of improvement, cohort 2 showed a positive but weaker relationship due to a relatively small number of patients (*n* = 5, Cond.R^2^ = 0.619, *p* = 0.222).

## Discussion

Three distinctive change patterns of brain activity were identified in SCC DBS based on longitudinal PET: (1) first brain changes induced by initial brief stimulation during implantation surgery, (2) early brain changes induced by short-term continuous therapeutic stimulation (1-month), and (3) late brain changes induced by long-term chronic continuous stimulation (6-months). Both DMN and SN showed initial rCBF changes at 1-month post-surgery. In addition, the DMN also showed the most robust long-term chronic stimulation effects, which were significantly correlated with clinical improvement. Importantly, the utilization of an alternative resting-state PET imaging technique involving [18 F]-FDG PET replicated the results obtained from [15 O]-water PET scans. Notably, this replication included the trajectory of DMN and highlighted their distinct changes based on the clinical outcomes. These findings provide novel evidence that SCC has differential time effects on brain changes in DMN, that might serve as a biomarker to track the clinical improvement in SCC DBS over time.

Depression can be defined as a network disorder associated with alterations in several interacting networks. More specifically, abnormal functional connectivity in the affective network (AN), FPN, DMN, and SN has been previously reported [26]. However, only a few studies have investigated the neural mechanisms underlying DBS for depression, with voxel-wise rather than network changes the focus of previously published reports [27–29]. Findings here suggest that SCC stimulation effects are primarily associated with functional brain structures in the SN and DMN.

Across networks previously implicated in depression pathophysiology and DBS mechanisms, SN and DMN rCBF decreased with the initial stimulation. Previous studies have demonstrated that repeated stimulation in the OR at the tractography-defined optimal target facilitates a sustained antidepressant effect for several weeks after surgery [8, 10, 12, 30], and is accompanied by decreases in SCC beta power measured using intraoperative recordings of local field potentials (LFP) [31]. Notably, several of the regions in the DMN and SN networks are structurally connected to the SCC target via the uncinate fasciculus (UF), cingulum bundle (CB), or forceps minor (FM), and stimulation of these pathways are necessary for the successful SCC DBS [7, 8]. Indeed, regions within the tractography-derived SCC-DBS network showed the same initial decrease of rCBF as the pre-defined SN and DMN ICNs, even though the SCC-DBS network was not significant after multiple comparison corrections. In addition, the same initial decrease of rCBF is also seen in the left anterior thalamus which are also structurally connected to the SCC target [7, 32, 33]. Therefore, an initial rCBF decrease in SN, DMN, and thalamus may reflect a network ‘***reset’*** in the directly connected regions to the SCC DBS target. Notably, post-operative brain activity decreases measured with [18 F]-FDG PET have also shown in patients with obsessive-compulsive disorder (OCD) receiving DBS to the bed nucleus of the stria terminalis (BNST) [34]. This apparent network ‘***reset***’ may be a necessary but insufficient change, as clinical improvement reverses without ongoing DBS, despite initial behavioral gains from initial OR testing [11]. In particular, the patients with higher initial reductions were associated with lower clinical improvements after surgery as the positive relationships in the DMN.

While the rCBF decreases in the SN were maintained with 1-month and 6-months chronic stimulation, the rCBF in DMN showed increases with chronic stimulation that were associated with clinical recovery. In addition, changes in depression severity scores were significantly associated with the changes of rCBF in DMN but were not correlated with other ICNs. Furthermore, responders who had a minimal reduction of 50% of the HDRS-17 from the baseline after 6-months of chronic stimulation showed a significantly increased rCBF with chronic stimulation in DMN, whereas non-responders did not. Interestingly, the switch in sign over time, including the initial decrease and late increase patterns, is consistent with a previous PET study examining the time course of antidepressant effects with medication in major depressive disorder (MDD) patients who showed a similar switch/non-switch pattern in responders and non-responders, respectively [14]. The slower and more progressive CBF and glucose metabolic changes seen with ongoing therapeutic stimulation are posited to reflect potential transsynaptic changes or activity dependent plasticity effects [35]. Such delayed activity changes seen with both CBF, and glucose metabolism PET may be a functional readout of such reported plasticity changes in model animals [35, 36]. Our DMN findings are consistent with previous reports of hyperconnectivity of the SCC with the DMN in TRD patients studied with rs-fMRI [37–39] and changes in DMN connectivity with various treatments [40, 41]. In addition, previous studies of OCD had reported the increased rCBF in DMN associated with reducing depressive symptoms when DBS was turned on, despite the different target including ventral capsule/ventral striatum (VC/VS) [33, 42]. Given the putative role of the DMN in maintaining internal mental states including, self-referential thoughts and actions [43–45], we have demonstrated that selective stimulation of frontal white matter induces a reproducible switch from interceptive to exteroceptive attention during intraoperative testing [10], consistent with first decreases in DMN seen in this study.

To further characterize which regions within the DMN were responsible for the differential initial decrease, and increase with chronic stimulation in responders, voxel-wise post-hoc analysis with default A was performed (Supplementary Fig. 4, *p* < 0.01). Our voxel-wise post-hoc analysis elucidated that anterior module in DMN, including medial prefrontal cortex (mPFC) regions, were associated with first brain changes. In contrast, posterior modules, including posterior cingulate cortex (PCC), were related to early and late changes. Previous studies reported that the DMN had been further divided into two functional subnetworks, the anterior and posterior subnetworks [46, 47]. Although there was an inconsistency in previous literature that has reported increased or decreased functional connectivity in DMN in patients with MDD [37, 48, 49], a recent voxel-wise meta-analysis revealed decreased connectivity in the posterior DMN and increased connectivity in the anterior DMN in patients with MDD [27]. Our group has demonstrated that responders to SCC DBS share unique streamlines from the stimulation site [7, 8], to the mPFC and dorsal anterior cingulate cortex (dACC), i.e., the anterior part of DMN, via the FM and CB streamlines, respectively [8]. Together, growing evidence suggests that the SCC DBS target rapidly impacted the anterior DMN, where the first brain change effects occur. In contrast, the PCC subnetwork slowly changed with chronic stimulation, and it may require neuroplastic changes beyond directly connected anterior DMN regions. Non-responders failed to show later DMN changes providing further support for this hypothesis with missing pathway activation to the mPFC via the UF and FM [7]. Unresolved is the apparent contradiction of a robust initial decrease in the anterior DMN but with a late increase in the same network, with the increases correlated with clinical effects. Quantitation of the baseline structural integrity of the targeted white matter bundles connecting these resting state network and longitudinal white matter changes with ongoing stimulation may help resolve this in future studies using next-generation DBS devices.

The present study has several strengths, including two independent cohorts, allowing us to test our hypotheses across two distinct resting state PET imaging modalities. Our results were consistent across the cohorts, despite minor differences in scanners, surgical targeting methods and exposure to varying amounts of intraoperative stimulation. CBF and glucose metabolism are expected to be coupled in a normal brain state and highly correlated with each other within subjects across brain regions (r > 0.7) [50]. This correlation has also been previously demonstrated in studies of SCC DBS studied with both glucose metabolism and CBF [1, 3]. As similarly shown here, responders and non-responders showed a common decreased SUVR with both tracers measured after surgery but diverged thereafter with chronic stimulation. These findings imply the possibility of generalizable imaging biomarkers of SCC DBS including studies of change network structural and functional connectivity using MRI [51], which allow us to track the clinical improvement over time. While cohort 2 had just one non-responder, additional research is essential to validate and establish the significance of these results. Our study has several limitations. First, the sample size was modest, with only 7 and 4 responders respectively, despite the significant statistical effects. As a result, significant effects were obtained after multiple comparisons correction for the main effects, but the post-hoc analyses were not corrected. In addition, it’s important to note that the limited sample size could potentially lead to an inflation of effect sizes. Future studies will be needed to enhance the statistical power of our findings. Second, our cohort 1 included patients with both unipolar and bipolar depression, although all patients were of comparable depressive severity, and showed comparable antidepressant effects with DBS. Notably, none developed hypomanic symptoms with ongoing stimulation regardless of their response status or dose changes. All patients were maintained on their baseline medications without changes throughout the entire 6-months. That said, there was not a standardized medication regimen or algorithm for patients in either cohort and all were on a wide variety of different medications. As discussed in the parent clinical paper [2, 4, 12], there were no differences in responders and non-responders as to the number of medication types. It is not possible to dissociate DBS effects from potential synergistic effects of medication. Similarly, there were differences in the DBS current dose used in responders and non-responders. Responders generally required lower doses as no changes were made if the patient was showing a steady decrease in depression symptoms over time. Non-responders on the other hand were the patients that had dose and eventually contact changes, but generally after the 6-month scan. No differences were found in the number of stimulation changes between the responder and non-responder during 6 months of active stimulation (χ^2^ tests, *p* = 0.949). As previously published, 6 of the 10 non-responders became responders by two years generally with adjustment of the contact being stimulated. While not controlled here, these subsequent observations are consistent with failure to see PET DMN changes in the 6-months non-responder subgroup. Third, our approach using the ICNs have both advantages and limitations. We adopted the ICNs as ROIs to examine the changes in rCBF or glucose metabolism. As functional connectivity analysis in ICNs was not performed, we were not able to exclude the possibility of changes in the functional network organization. Nevertheless, our findings have successfully replicated the previous voxel-wise two PET studies that showed the changes in areas associated with the DMN [1, 3]. Moreover, a previous fMRI study has also shown that the functional activity in several areas related to DMN decreases when SCC DBS is turned on, similar to our acute change [52]. Interestingly, it is notable that the lateral frontal clusters within Default A including dorsolateral prefrontal cortex (dlPFC) increases seen with stimulation in responders (Supplementary Fig. 4) were located near the classic transcranial magnetic stimulation (TMS) target used to treat depression, defined using SCC functional connectivity methods [53]. To improve the analysis accuracy considering the group-specific functional connectivity, we generated a group-specific DMN template for cohort 1 data using 14 patients with currently usable resting-state fMRI data. The seed location was set at the PCC area, which is the pivotal component of DMN (x = ±7, y = –43, z = +33) [54]. Obviously, the PCC functional connectivity map (green) is highly overlapped with the Default A (yellow) from previous study [15], and the results also showed a similar trajectory of rCBF changes in Default A (Supplementary Fig. 5). Use of large ROIs that span the brain may dilute small significant effects detectable using voxel-wise analyses. We utilized the post-hoc voxel-wise analyses to overcome this issue but did not perform a comparable analysis for the other significant ICNs, as none correlated with clinical outcomes. Finally, there is a clear noise signal in the PET scans around the DBS lead. In addition, we also note that the distortion can appear in warping to the MNI due to the limitations of PET images. We used a conservative approach to eliminate any spurious attributions of decreases to areas with low signal drop out or partial volume effects by use of an individual reversed electrode mask, accommodating the unique trajectory of lead implantation for each subject. Future studies should work to improve recovery of signal near the electrode. Despite this limitation which would most prominently impact the SCC and anterior DMN changes, the artifact could not explain the posterior DMN effects. Further, artifacts would magnify the decreases but could not explain the increase that are associated with clinical response.

In summary, the trajectory of brain changes with SCC DBS is not linear, consistent with the chronology of therapeutic effects. The present data support the notion that the DMN resets with initial stimulation but undergoes more complex plastic effects with chronic DBS. As such, DMN activity may serve as useful biomarker to track the clinical improvement with SCC DBS.

### Supplementary information

Supplementary Material

## Data Availability

The data that support the findings of this study are available from the corresponding author, KC, upon reasonable request.
