# Supplementary material for: Whole brain network effects of subcallosal cingulate deep brain stimulation for treatment-resistant depression
Source: Mol Psychiatry. 2023 Nov 2;29(1):112–20. doi: 10.1038/s41380-023-02306-6 (PMC11078711; doi:10.1038/s41380-023-02306-6)

**Supplementary Figure 1.** Diagram for cerebral blood flow (CBF) positron emission tomography (PET) imaging data processing procedure with T1-weighted anatomical and computed tomography (CT) images.


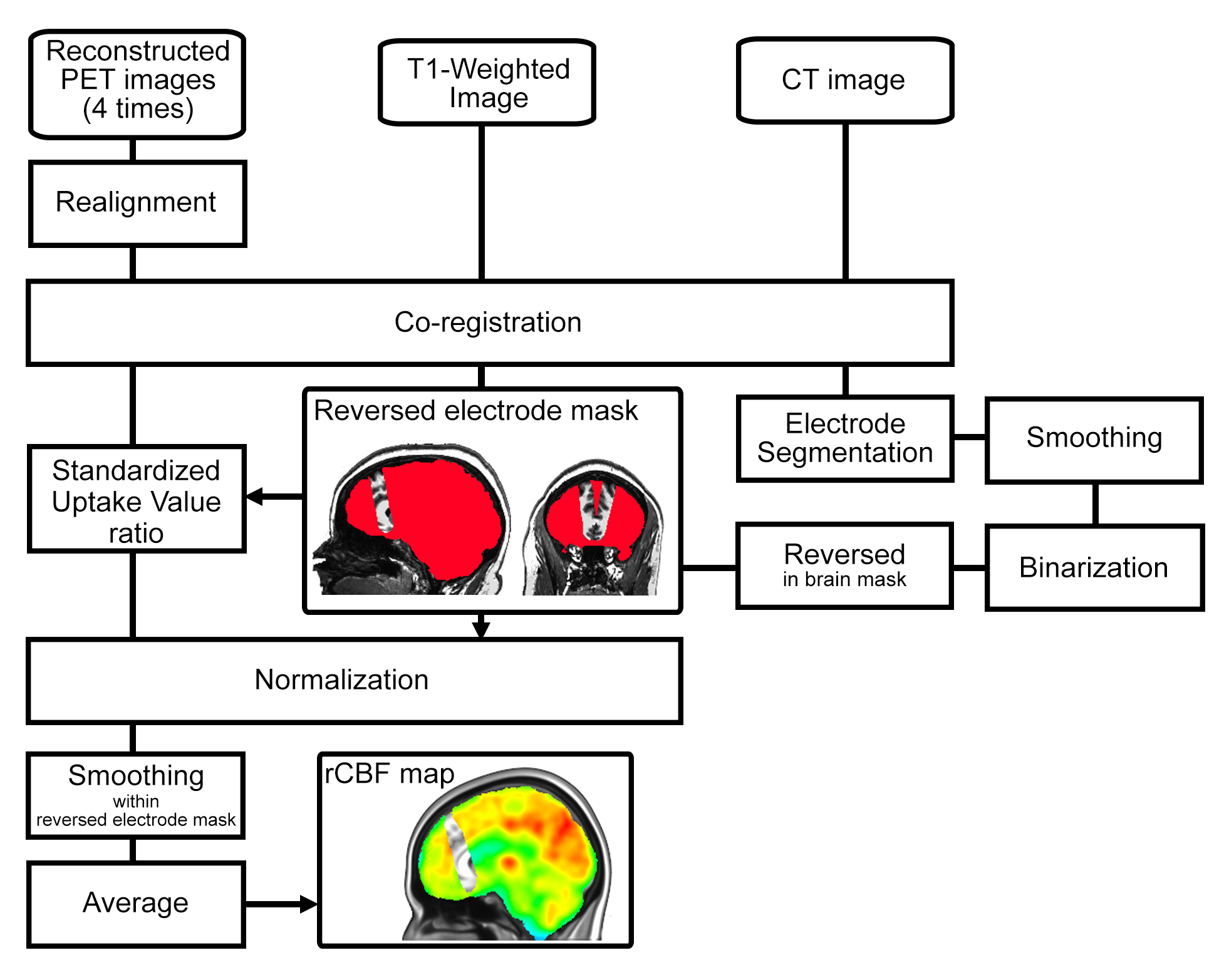


**Supplementary Figure 2**. Changes of rCBF over time with deep brain stimulation (DBS) in treatment resistant depression (TRD) patients. (A) Changes of rCBF in 18 networks, including 17 intrinsic connectivity networks (ICNs) from Yeo et al., 2011 and area connected to the subcallosal cingulate cortex (SCC) using diffusion tensor imaging (DTI), namely SCC-DBS network (Hot Pink). (B) SCC-DBS network showed significant differential trajectory of CBF over time (F=4.79, p=0.005). In addition, post-hoc analysis also showed rapid changes and maintained over time. A SCC-DBS network template was defined by dilated mean WM connectivity from SCC using diffusion tensor imaging (DTI). Mapping results of the SCC-DBS networks onto the surface was shown in (B). Asterisk represents statistically significant changes (*: p<0.05, **: p<0.005).


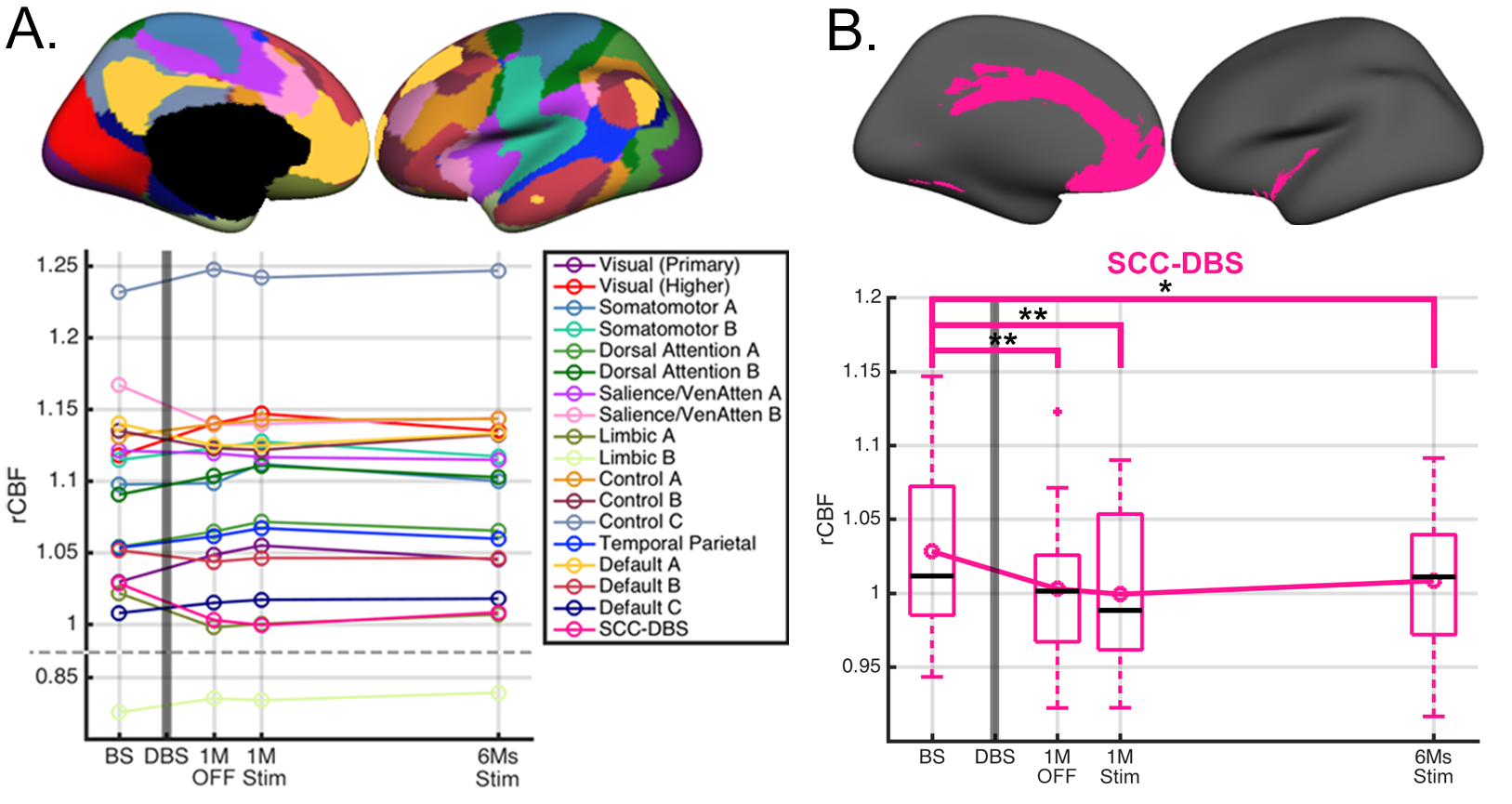


**Supplementary Figure 3**. Longitudinal changes of rCBF in the default A between responders and non-responders in (A) unipolar and (B) bipolar depression. Blue represents the responders, while red represents the non-responders. In unipolar depression, there are five responders and five non-responders, whereas in bipolar depression, there are two responders and five non-responders. The second scan of one non-responder in unipolar depression and one responder in bipolar depression was excluded due to poor image quality. Transparent lines in the boxes represent individual longitudinal trajectories, and the solid line is the mean of longitudinal trajectory.


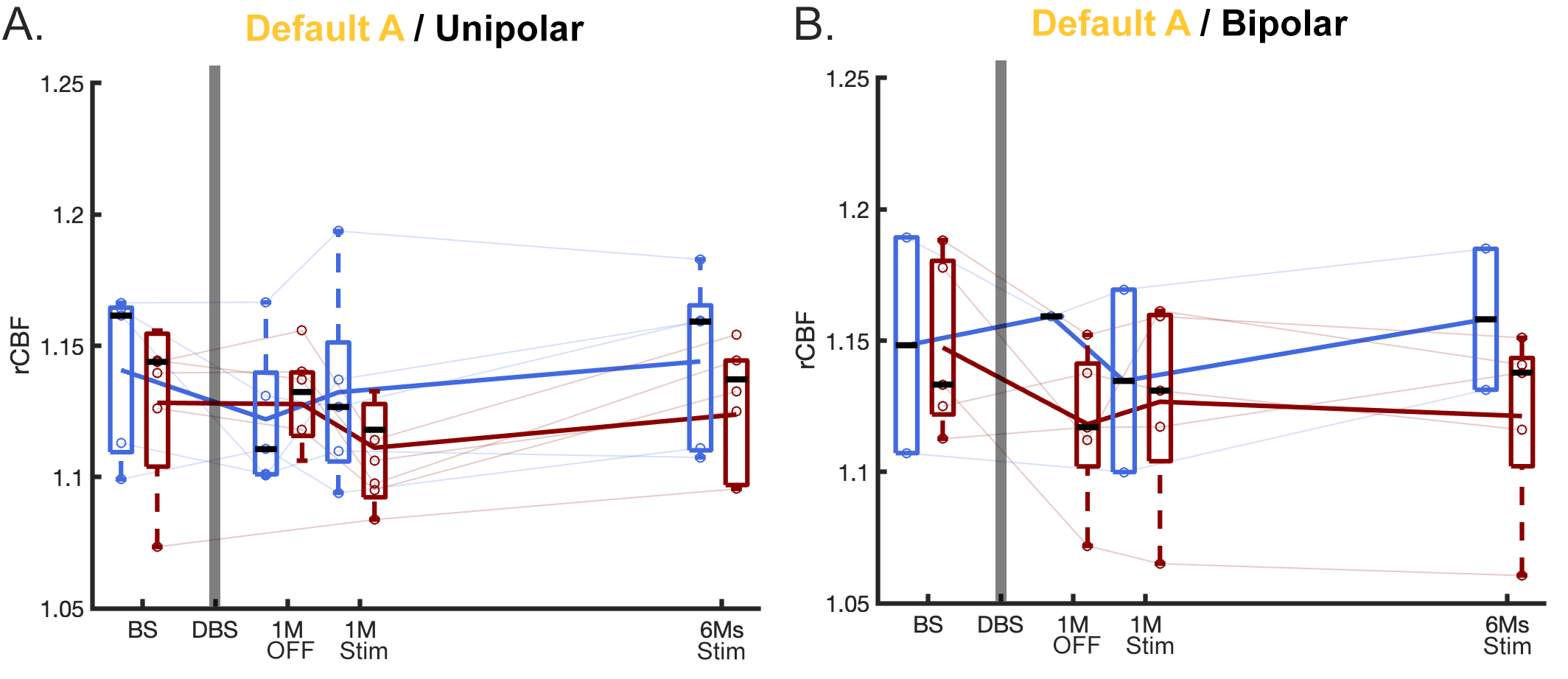


**Supplementary Figure 4**. Voxel-wise post-hoc contrast tests in changes after surgery or the chronic stimulation effects, additionally early and late stimulation effect, within default A in responders (p<0.01). (A) Decreased rCBF after surgery was represented by the purple color (Scan 2 vs. Scan 1), while increased rCBF due to the stimulation effects between post-DBS surgery and 6-month chronic stimulation was denoted by the green color (Scan 4 vs. Scan 2). (B) The orange color indicated the increased rCBF of early stimulation effect between post-DBS surgery and 1-month chronic stimulation (Scan 3 vs. Scan 2), while the red color represented the increased rCBF of late stimulation effect between 1-month and 6-month chronic stimulation (Scan 4 vs. Scan 3).


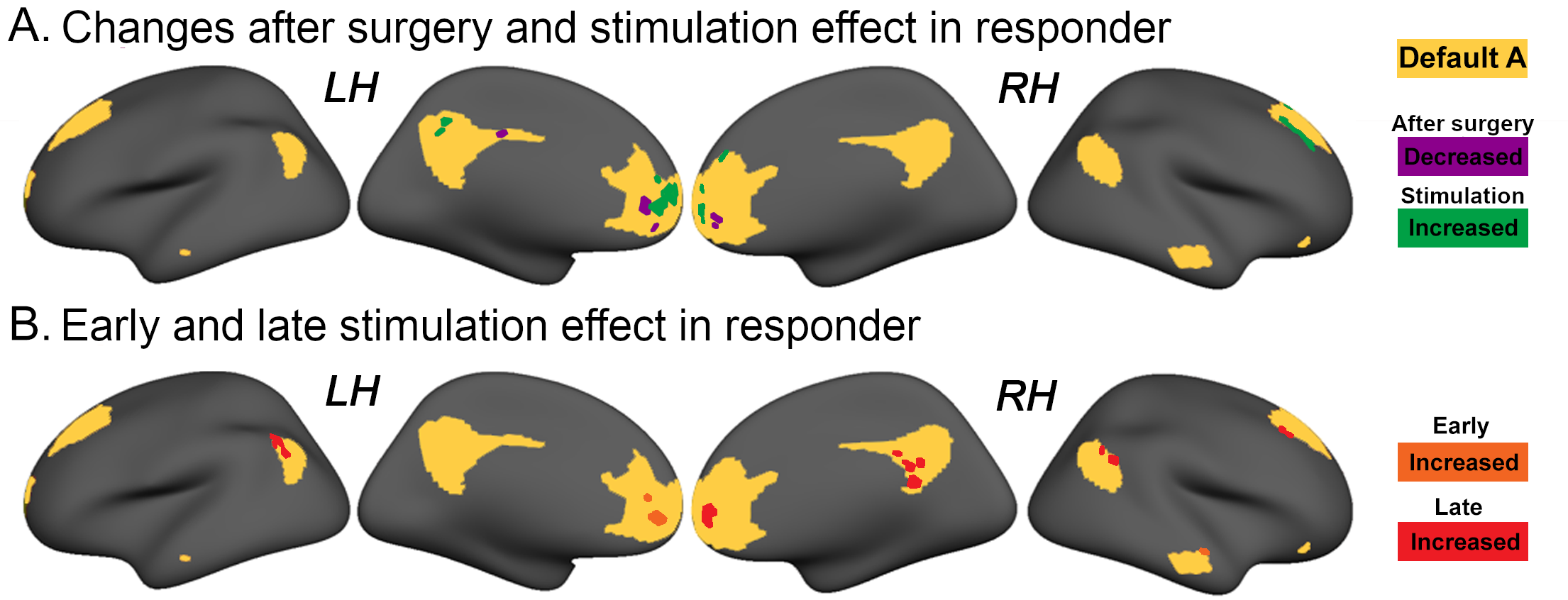


**Supplementary Figure 5**. Replicated results in the posterior cingulate cortex (PCC) functional connectivity (FC) map using the cohort 1 resting-state fMRI (n=14). (A) anatomical locations of Default A clusters in Yeo’s template (yellow) and PCC FC (green) on the cortical surface. Viewing the anatomical locations reveals that the yellow regions corresponding to Default A were highly overlapped with the green regions corresponding to PCC FC. (B) a significant time effect of rCBF in the PCC FC using a linear mixed model for repeated measure (p=0.024). For post-hoc analysis, there were not only early decreased changes of rCBF after surgery (Scan 2 vs. Scan 1: p=0.010, and Scan 3 vs. Scan 1: p=0.025), but also late increased rCBF changes between after surgery and 6-months stimulation (p=0.037). (C) Changes of rCBF in the PCC FC in the responders (blue) and the non-responders (red). In the responders, there were not only early decreased changes of rCBF after surgery (p=0.033), but also increased rCBF changes between after surgery and 6-months stimulation (p=0.003) and between 1-month chronic stimulation and 6-month chronic stimulation (p=0.036). In the non-responders, there were early decreased changes of rCBF after surgery (p=0.048) and maintained over time. Asterisk represents statistically significant changes (*: p<0.05, **: p<0.005). Transparent lines in the boxes represent individual longitudinal trajectories, and the solid line is the mean of longitudinal trajectory.


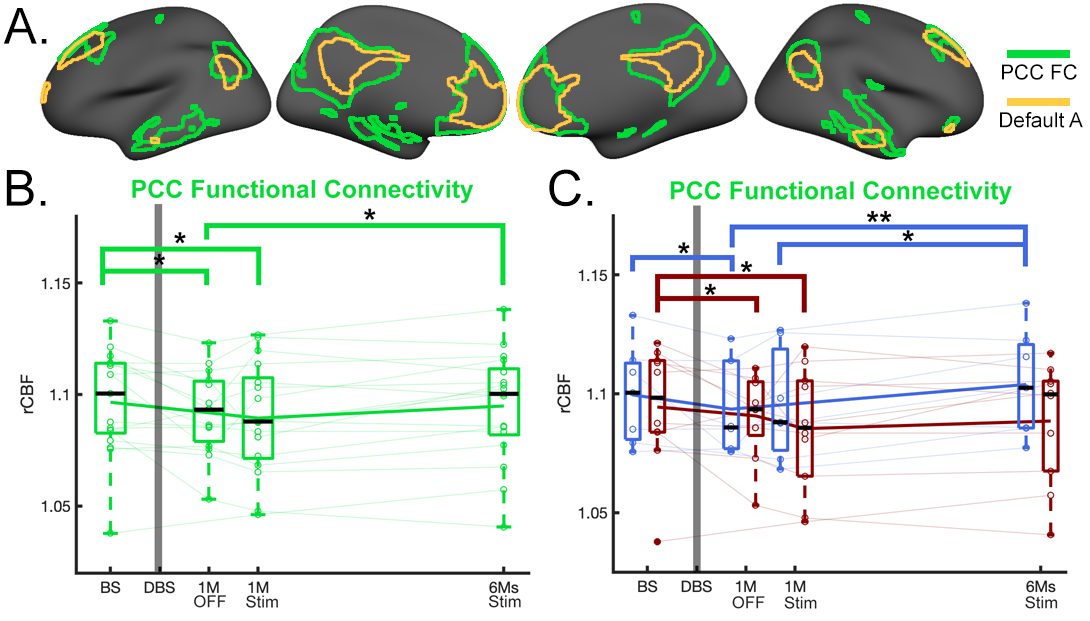

Supplement: Supplementary file 1 — Supplementary Material [file 41380_2023_2306_MOESM1_ESM.docx]
